# Multi-voxel pattern analysis in human hippocampal subfields

**DOI:** 10.3389/fnhum.2012.00290

**Published:** 2012-10-18

**Authors:** Heidi M. Bonnici, Martin J. Chadwick, Dharshan Kumaran, Demis Hassabis, Nikolaus Weiskopf, Eleanor A. Maguire

**Affiliations:** ^1^Wellcome Trust Centre for Neuroimaging, Institute of Neurology, University College LondonLondon, UK; ^2^Institute of Cognitive Neuroscience, University College LondonLondon, UK; ^3^Gatsby Computational Neuroscience Unit, University College LondonLondon, UK

**Keywords:** MVPA, hippocampus, subfields, scenes, memory, fMRI, CA3, DG

## Abstract

A complete understanding of the hippocampus depends on elucidating the representations and computations that exist in its anatomically distinct subfields. High-resolution structural and functional MRI scanning is starting to permit insights into hippocampal subfields in humans. In parallel, such scanning has facilitated the use of multi-voxel pattern analysis (MVPA) to examine information present in the distributed pattern of activity across voxels. The aim of this study was to combine these two relatively new innovations and deploy MVPA in the hippocampal subfields. Delineating subregions of the human hippocampus, a prerequisite for our study, remains a significant challenge, with extant methods often only examining part of the hippocampus, or being unable to differentiate CA3 and dentate gyrus (DG). We therefore devised a new high-resolution anatomical scanning and subfield segmentation protocol that allowed us to overcome these issues, and separately identify CA1, CA3, DG, and subiculum (SUB) across the whole hippocampus using a standard 3T MRI scanner. We then used MVPA to examine fMRI data associated with a decision-making paradigm involving highly similar scenes that had relevance for the computations that occur in hippocampal subfields. Intra- and inter-rater scores for subfield identification using our procedure confirmed its reliability. Moreover, we found that decoding of information within hippocampal subfields was possible using MVPA, with findings that included differential effects for CA3 and DG. We suggest that MVPA in human hippocampal subfields may open up new opportunities to examine how different types of information are represented and processed at this fundamental level.

## Introduction

The hippocampus is composed of a number of subregions which were named CA1, CA2, and CA3 by Lorente De No (1934). These subfields are adjoined by neighbouring areas the dentate gyrus (DG), the subiculum, presubiculum, parasubiculum, and entorhinal cortex, to form the extended hippocampal formation (Amaral and Lavenex, 2007). Studies in rodents (e.g., Kesner et al., 2004; Leutgeb et al., 2004, 2007; Leutgeb and Leutgeb, 2007; Alvernhe et al., 2008; Hunsaker and Kesner, 2008; Gilbert and Brushfield, 2009; Aimone et al., 2011) and computational models (Marr, 1971; Treves and Rolls, 1994; McClelland et al., 1995; Rolls, 2010; O'Reilly et al., 2011) suggest that computations that are key to episodic memory, such as pattern separation and pattern completion, occur in specific regions within the hippocampal formation. Pattern separation is the process of distinguishing similar memories from each other and is thought to occur in DG and CA3. Pattern completion concerns the retrieval of previously stored memories from partial cues and is thought to involve CA3.

In humans, examination of these regions *in vivo* has proved difficult, but advances in high-resolution structural and functional MRI have begun to make it possible to localise fMRI BOLD activity to specific hippocampal subfields with greater confidence (e.g., Zeineh et al., 2000a,b, 2001, 2003; Bakker et al., 2008; Small et al., 2011; Suthana et al., 2011; Duncan et al., 2012; see Carr et al., 2010 for a review). fMRI studies published to date that reported hippocampal subfield findings typically employed a standard mass-univariate approach to data analysis. In the last number of years there has been increasing interest in alternative methods that exploit the intrinsically multivariate nature of fMRI data. The motivation for this change stems from the belief that there may be information present in the distributed pattern of activation across voxels that is missed when looking at each voxel independently as in the mass-univariate method (Haynes and Rees, 2006; Norman et al., 2006; Mur et al., 2009; Pereira et al., 2009; Chadwick et al., 2012; Rissman and Wagner, 2012). This type of multivariate approach is commonly known as multi-voxel pattern analysis (MVPA), or “decoding”.

It has been possible to decode specific spatial locations within a virtual environment from patterns of activity across voxels in the hippocampus (Hassabis et al., 2009; Rodriguez, 2010). Similarly, Chadwick et al. (2010) were able to predict which episodic memory participants were recalling from patterns of activity across voxels in the hippocampus moreso than neighbouring entorhinal and parahippocampal cortices, even when memories were highly overlapping (Chadwick et al., 2011). Because MVPA allows us to examine individual memory representations, use of this technique could open up new opportunities to examine hippocampal representations in terms of their content, and how they might change over time, with aging, and pathology (see Chadwick et al., 2012 for a review). Given the potential of MVPA and the importance of understanding the functional contributions of specific subregions within the hippocampal formation, it would seem advantageous to combine the two.

In order to do this effectively, we (1) wanted to include the whole hippocampus and (2) to separate, as far as possible, each individual subregion from the others, to examine their specific contributions. (3) While many studies report high in plane resolution in their MRI scans (e.g., 0.39 × 0.39 mm—Zeineh et al., 2000a,b), this is often acquired in thick slices (e.g., 3 mm). The skewed resolution from non-isotropic voxels distorts delineation of subfields (making it particularly difficult in anterior hippocampal regions), which cannot be overcome by spatial interpolation. We therefore wanted to acquire data with isotropic voxels to circumvent these issues and further minimise resampling artifacts when co-registering the datasets. It should also be noted that in using a searchlight MVPA procedure (as we do here—see “Materials and Methods”; see also Hassabis et al., 2009; Chadwick et al., 2010, 2012), the use of unfolding and flat-mapping to visualise activation in the subfields (e.g., Zeineh et al., 2000a) is not suitable because local patterns of activity among clusters of voxels get disrupted if data are projected from 3D to 2D flat maps (Carr et al., 2010).

Examining the literature for methods of delineating subregions of the hippocampal formation, it is surprising how the criteria outlined above prove difficult to satisfy. Numerous methods have been described, but none has achieved wide acceptance. While an exhaustive review of extant methods is beyond the scope of this paper, we summarize the main issues as they relate to the aim of our study. First, some methods do not in fact examine the whole hippocampus. Some restrict their analysis to a few slices of the hippocampus (Mueller et al., 2007) or just 1 cm of the structure (e.g., Mueller et al., 2010), others do not delineate subfields within the head of the hippocampus (Zeineh et al., 2000a,b, 2001, 2003; Eldridge et al., 2005; Ekstrom et al., 2009; Suthana et al., 2009; Preston et al., 2010), or its tail (Zeineh et al., 2000a,b, 2001, 2003; Eldridge et al., 2005), while others focus only on the body of the hippocampus (Yushkevich et al., 2010), or on one or two specific subfields (Moreno et al., 2007; Bartsch et al., 2011).

Second, aside from consideration of whether the whole hippocampus is available for subregion analysis, only two studies report being able to delineate CA2 (Malykhin et al., 2010; Yushkevich et al., 2010). In both cases high field scanners were employed (4T and 4.7T, respectively), thus identifying CA2 with confidence likely remains beyond the capability of studies using standard 3T scanners. More seriously, most methods do not have sufficient resolution or contrast to separate CA3 from DG (e.g., Zeineh et al., 2000a; Eldridge et al., 2005; Kirwan and Stark, 2007; Bakker et al., 2008; Carr et al., 2009; Ekstrom et al., 2009; Cho et al., 2010; Mueller et al., 2010; Preston et al., 2010). Functional differentiation within the hippocampus, be that down its long axis (e.g., Moser and Moser, 1998; Maguire et al., 2000; Fanselow and Dong, 2010; Poppenk and Moscovitch, 2011), or within the subfields (Marr, 1971; Treves and Rolls, 1994; McClelland et al., 1995; Leutgeb et al., 2004; Leutgeb and Leutgeb, 2007; Hunsaker and Kesner, 2008; Gilbert and Brushfield, 2009; O'Reilly et al., 2011) is well-established. Not being able to examine the anterior and posterior portions of the hippocampus, or being unable to distinguish the roles of CA3 and DG, limits the scope of studies and the conclusions that can be drawn.

A third issue concerns how delineation is achieved. Most of the papers cited above manually segmented the subregions. This is time-consuming and ideally involves at least two operators in order to test the reliability of segmentation (although many studies do not report any reliability measures). Two main automated procedures have been reported. Operating at 4T and with its main focus the evaluation of clinical scans, Yushkevich et al's (2010) segmentation procedure was able to delineate CA1, CA2, CA3, DG, and subiculum. While seeming to achieve accurate subfield segmentation, unfortunately, as noted above, it was not possible to identify subfields in the head and tail of the hippocampus, only in the body, currently limiting the utility of this approach outside of the clinical domain. The other automated procedure for segmenting hippocampal subfields is available as part of the Freesurfer analysis programme (Fischl et al., 2002, 2004). The initial development of this procedure (Van Leemput et al., 2009), and the basis of its current implementation, is the manual subfield segmentation of the right hippocampi of 10 individuals ranging in age from 22 to 89 years, where data were acquired at high resolution (0.38 × 0.38 × 0.8 mm) and averaged over five scans to achieve higher signal-to-noise ratio (SNR). The definitions of the boundaries of the subfields are very different from other protocols (e.g., Carr et al., 2009; Malykhin et al., 2010; Yushkevich et al., 2010) and do not seem to correspond to delineations from previous studies or indeed from atlases of hippocampal anatomy (e.g., Duvernoy, 2005); instead the delineations were based on geometrical rules. The authors provide no rationale for the use of these specific boundaries and cite no previous references using a similar protocol. In addition, how accurately their procedures generalise to scans acquired with lower resolution and SNR (as in Hanseeuw et al., 2011; Teicher et al., 2012) is also unknown.

It is evident that delineation of hippocampal formation subregions, a prerequisite for our research question, remains a substantial challenge (Van Strien et al., 2012). We considered the automated procedures as yet to incomplete (Yushkevich et al., 2010) or inexact (Van Leemput et al., 2009) for our purpose. Instead, we devised the following protocol to achieve our aims: using a standard clinical 3T whole-body MRI scanner, for each participant we acquired a set of high-resolution T2-weighted structural scans (0.5 mm isotropic voxels—see “Materials and Methods” for details) which allowed us to increase subfield boundary contrasts, permitted manual subfield segmentation within the whole hippocampus including head and tail, and the ability to identify the subiculum, CA1, and separate CA3 from DG (CA2 could not be separated and was included with CA3) guided by the Duvernoy (2005) hippocampus atlas and other resources (see “Materials and Methods”).

Having established a means of identifying hippocampal subregions that was suitable for our purpose, we next required a task for participants to perform during high-resolution fMRI. Bonnici et al. (2012) used MVPA to investigate the role of the hippocampus in pattern separation and pattern completion in a simple decision-making task involving two highly similar scenes. They found that more distinct representations of the scenes were present in the hippocampus compared to entorhinal and parahippocampal cortices, consistent with its role in pattern separation. When they examined morphed scenes that spanned a continuum between the original two scenes, they found evidence for pattern completion in the hippocampus. These hippocampal findings clearly prompt further questions about what might be occurring within hippocampal subfields during this task.

Given that sets of high-resolution T2-weighted structural scans were available for the participants in the Bonnici et al. (2012) study, we set out to identify CA1, CA3, DG, and SUB (for convenience referred to hereafter as “subfields”) using those scans, and then re-analyzed the fMRI data from that study this time focusing our MVPA analyses on the hippocampal subfields. In so doing, our main aims were to test the viability of our subfield segmentation procedure and the feasibility of conducting MVPA analyses in the hippocampal subfields. In this study, therefore, we were primarily concerned with ascertaining if above-chance levels of decoding were possible within the hippocampal subfields, and whether findings, if any, were consistent with the mechanisms proposed to be at work there. Specifically, when we are exposed to a stimulus, pattern separation, purportedly driven by the DG, leads to the formation of a unique, orthogonalized representation within CA3. These distinct traces can be retrieved when a cue triggers completion of the original CA3 activity pattern (pattern completion), which in turn drives CA1, from where the entire distributed cortical memory trace can be reactivated (Marr, 1971; Treves and Rolls, 1994; McClelland et al., 1995; Rolls, 2010; O'Reilly et al., 2011). Thus, we wondered whether decoding within CA3 and CA1 might feature prominently in our results.

## Materials and methods

### Participants

There were 16 healthy right-handed participants (8 male, mean age 24.4 years, SD 2.8, range 21–30) who had taken part in the Bonnici et al. (2012) study. All had normal or corrected-to-normal vision. Informed written consent was obtained from each participant in accordance with the approval of the University College London research ethics committee, and the Declaration of Helsinki, and is archived by the authors.

### Stimuli and task

**Figure 1:**
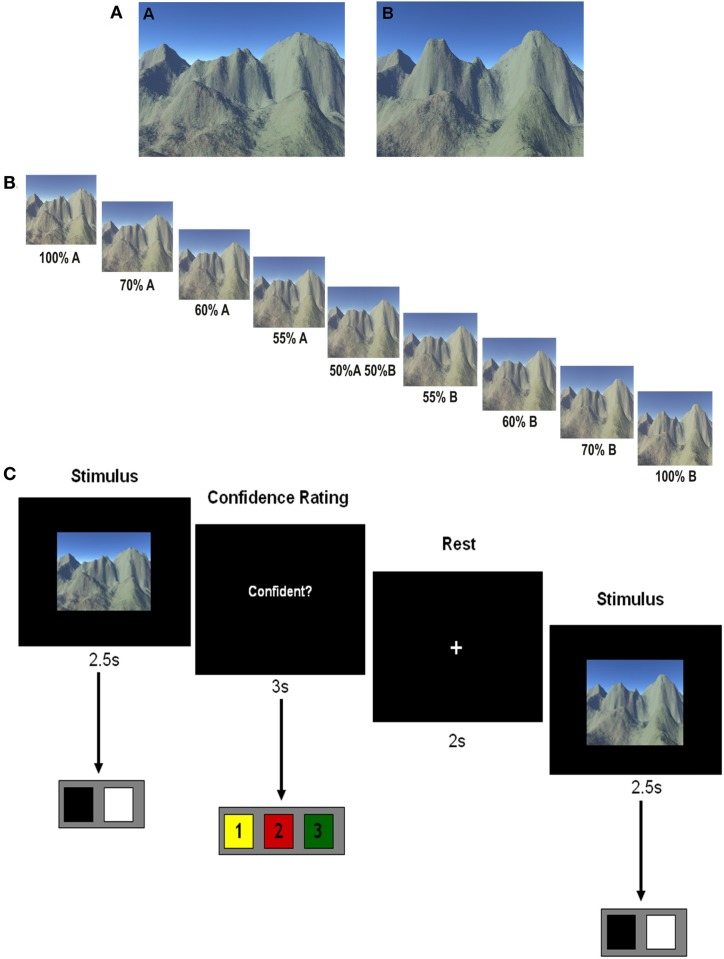
**The stimuli and experimental task. (A)** The two original scenes—note they were not labeled A and B in the actual experiment. **(B)** The morph continuum proceeding from 100% scene A to 100% scene B. **(C)** A timeline of an example single trial with stimulus duration of 2.5 s during which the participant registered their decision. Participants then indicated their confidence in that decision during the next 3 s from a choice of “not sure,” “fairly sure,” and “very sure”. There was a 2 s rest period before the next trial.

Full details are provided in Bonnici et al. (2012), with the key points reprised here for convenience. Two scenes; scene A and scene B (Figure 1A), were created using Terragen, version 0.9.43 for Windows (www.planetside.co.uk). Scene A was created first, and then modified to create scene B. Several phases of piloting ensured that the two scenes were regarded as highly similar whilst being distinct and were approximately equated for the number of constitute elements and overall complexity. Once the two scenes were created, seven morphed scenes were generated using Morph Age, version 4 for Mac (www.creaceed.com/morphage). Seven morphs were generated to proceed in a continuous fashion from scene A to scene B (70% A and 30% B, 60% A and 40% B, 55% A and 45% B, 50% A and 50% B, 45% A and 55% B, 40% A and 60% B, 30% A and 70% B). As the morph levels approached 50%, more features from the two original stimuli become shared, increasing the ambiguity (Figure 1B).

Participants were aware that in the experiment they would receive a monetary reward for their correct answers, while wrong answers lost money. In a training session prior to scanning, participants learnt which action was rewarded (e.g., action A-right button press) in relation to a given stimulus (i.e., scene A). The two scenes that were employed (scene A, scene B) were never labeled as such during the experiment. During this phase, participants were presented with scene A or B one at a time each for 2.5 s. Allocation of button press was switched for half of the participants. In each trial they were given feedback informing them if their choice was correct or incorrect. To ensure that choice performance had stabilized before scanning, each participant performed at least 20 trials during this phase, although all reached criterion (10 correct responses in a row) well before this (see “Results”).

In the next phase of the pre-scan training session, the morph stimuli as well as the original scene stimuli were presented in pseudo-random order, each scene shown for 2.5 s, and three times during the course of the training session. Once again participants were instructed to choose the action most likely to yield reward given the composition of the scene being viewed; no feedback was given. Following each trial they were asked to provide a confidence rating about the choice they had just made: 1 = not sure, 2 = fairly sure, and 3 = very sure. After this learning phase that included the original scenes and the morphs, participants then repeated phase one, viewing the original two scenes again to ensure behavioural performance was stabilized before scanning.

During scanning, participants saw the two scenes, 100% A and 100% B, as well as the seven morphed stimuli one at a time in a pseudo-random order ensuring there were no biases toward either scene A or B (see example trial timeline in Figure 1C). Stimuli were presented 40 times each. As before, participants were instructed to choose the action most likely to yield reward, given the composition of the scene being viewed, and then to provide a confidence judgement. No feedback was given during the scanning phase of the experiment, although participants were instructed that they would be paid in proportion to their performance on the task at the end of the experiment. In the analysis where the original scenes were compared, trials where the participant did not make a decision were excluded from the MVPA analysis, as were decisions that were incorrect, and decisions that were rated as “not sure” (on average 9% of trials were excluded). In the analysis involving the 50% morph scene, trials where the participant did not make a decision were excluded from the MVPA analysis, as were decisions that were rated as “not sure” (there was no right or wrong answer for the 50% morph scene; on average 24% of trials were excluded).

After scanning, each participant was debriefed. They were first asked to perform a probe test, where 40 stimuli were presented in the same format as the scanning task. Stimuli consisted of 20 scenes based on 100% scene A and 20 based on 100% scene B. In each case the stimulus was exactly the same as the original scene, but with successive shifts in view angle of 5°, either to the right or the left. Altogether there were 10 scene A stimuli shifted to the right, 10 shifted to the left, and 10 scene B stimuli shifted to the right, 10 shifted to the left. The aim of this task was to explore the nature of the strategies used during the discrimination task. If participants were able to select the correct action in response to rotated versions of the original scenes this would suggest that behavioral performance was based on view-independent scene representations, rather than the sampling of individual features. Finally, each participant was asked to draw what he/she could remember of the two scenes (100% A and 100% B).

### MRI acquisition

High-resolution structural images were acquired on a 3T whole body MRI scanner (Magnetom TIM Trio, Siemens Healthcare, Erlangen, Germany) operated with a radiofrequency (RF) transmit body coil and 32-channel head RF receive coil. Imaging was limited to a partial volume focused on the temporal lobes. A single-slab 3D T2-weighted turbo spin echo sequence with variable flip angles (SPACE; Mugler et al., 2000) in combination with parallel imaging was employed to simultaneously achieve a high image resolution of ~500 μm, high sampling efficiency and short scan time while maintaining a sufficient SNR. After excitation of a single axial slab the image was read out with the following parameters: resolution = 0.52 × 0.52 × 0.5 mm^3^, matrix = 384 × 328, partitions = 104, partition thickness = 0.5 mm, partition oversampling = 15.4%, field of view = 200 × 171 mm^2^, echo time (TE) = 353 ms, repetition time (TR) = 3200 ms, parallel imaging with GRAPPA × 2 in phase-encoding (PE) direction, bandwidth = 434 Hz/pixel, echo spacing = 4.98 ms, turbo factor in PE direction = 177, echo train duration = 881, averages = 1.9. For reduction of signal bias due to, e.g., spatial variation in coil sensitivity profiles, the images were normalized using a prescan and a weak intensity filter was applied as implemented by the scanner's manufacturer. To improve the SNR of the anatomical image, four scans were acquired for each participant, co-registered and averaged. It took 12 min to obtain each scan with a total scanning time of 48 min. In addition, a whole brain 3D FLASH structural scan was acquired with a resolution of 1 × 1 × 1 mm.

High-resolution functional MRI scans were acquired in a partial volume focused on the temporal lobes. A 3T Magnetom Allegra head only MRI scanner (Siemens Healthcare, Erlangen, Germany) operated with the standard RF transmit-receive head coil was used to acquire the functional data with a T2^*^-weighted single-shot echo-planar imaging (EPI) sequence (in-plane resolution = 1.5 × 1.5 mm^2^; matrix = 128 × 128; field of view = 192 × 192 mm^2^; 35 slices acquired in interleaved order; slice thickness = 1.5 mm with no gap between slices; TE = 30 ms; asymmetric echo shifted forward by 26 PE lines; echo spacing = 560 μs; TR = 3.5 s; flip angle α = 90°). All data were acquired at 0° angle in axial orientation with PE in the anterior–posterior direction. An isotropic voxel size of 1.5 × 1.5 × 1.5 mm was chosen for an optimal trade-off between BOLD sensitivity and spatial resolution. Further, the isotropic voxel dimension reduced re-sampling artefacts when applying motion correction. To ensure optimal data quality, images were reconstructed online and underwent online quality assurance (Weiskopf et al., 2007). For distortion correction (Hutton et al., 2002), field maps were acquired with a standard manufacturer's double echo gradient echo field map sequence (TE = 10.0 and 12.46 ms, TR = 1020 ms; matrix size = 64 × 64), using 64 slices covering the whole head (voxel size 3 × 3 × 3 mm). Scanning was performed in a single session and took approximately 45 min.

### Hippocampal subfield segmentation

Manual segmentation of CA1, CA3, DG, and SUB was performed with the ROI module of the Anatomist software (http://brainvisa.info/index.html) on the averaged T2-weighted high-resolution structural image of each participant. Segmentation was performed primarily using the Duvernoy (2005) hippocampus atlas as a guide, with West and Gundersen (1990) and Mai et al. (2008) as additional resources. Because these guides describe segmentation with 3 mm thick slices, and our slices were 0.5 mm thick, post-mortem data described by Yushkevich et al. (2009) acquired at 9.4T and using slices of ~0.2 mm were used as an additional reference.

**Figure 2:**
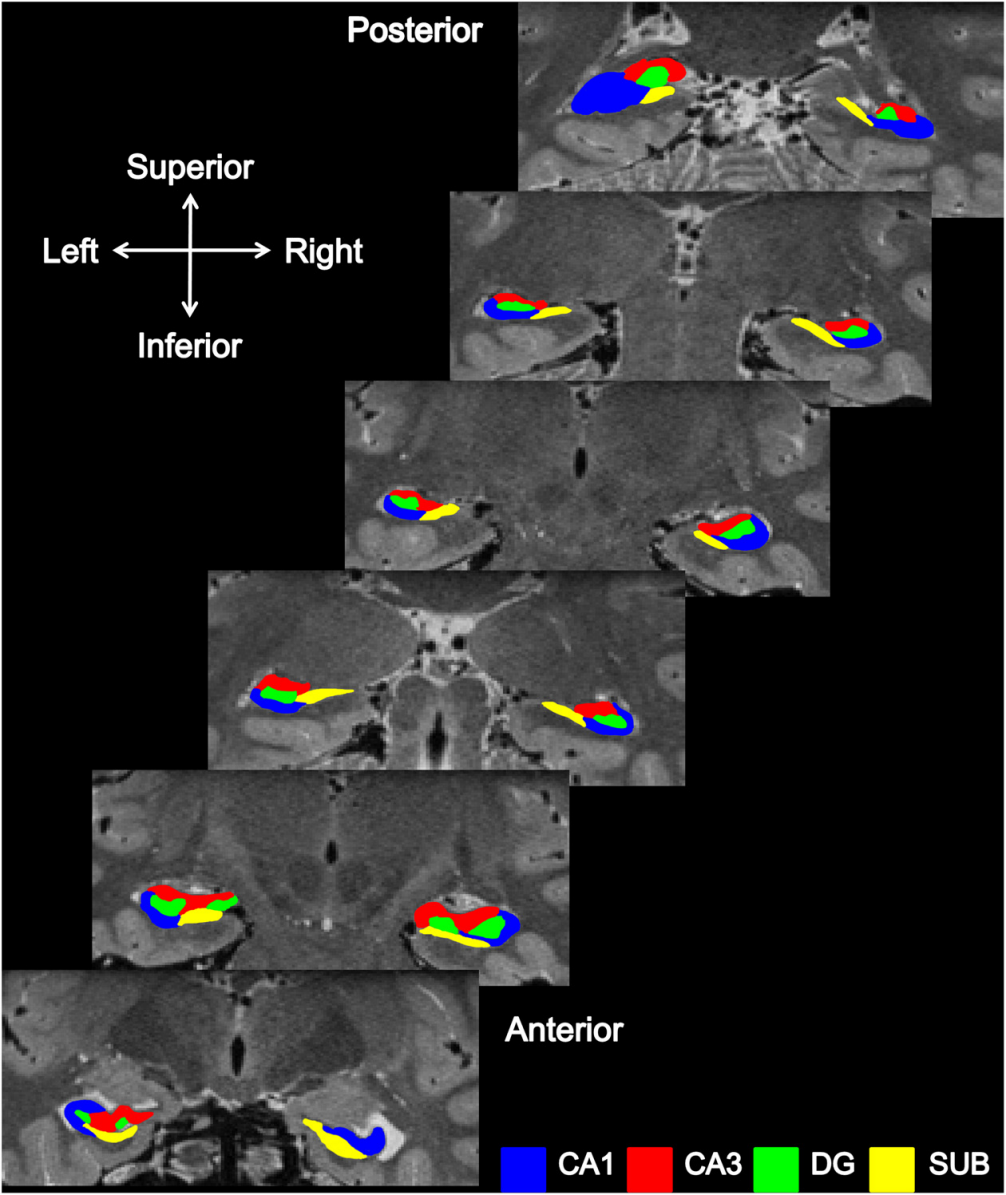
**Subfield segmentation in the coronal plane.** Coronal sections through an averaged T2-weighted image of both the left and right hippocampus of a participant.

Segmentation was first performed in the coronal view, one subfield at a time, starting with DG, then CA1, CA3, and finally SUB (Figure 2). The starting point for segmentation was the slice where the body emerged from the head of the hippocampus (anterior, Figure 2), distinguished as the place where the fimbria detaches from the head of the hippocampus, as described in Duvernoy (2005), and working backwards through the body toward, but not including, the tail of the hippocampus. No attempt was made to separate the presubiculum and parasubiculum from the SUB proper and so both were included in the SUB subfield. This region links the hippocampus to the entorhinal area medially and adjoins to CA1 laterally. CA1 continues from the SUB and ends once the curve (genu) of the Cornu Ammonis (CA) is reached. The division between CA1 and CA3 was identified with a narrowing of the CA when viewed coronally. The hippocampal sulcus provided a distinguishing boundary between DG and CA1 and CA3.

Once segmentation of the hippocampal body was completed, segmentation of the head and tail of the hippocampus were conducted in turn. Coronally, the head of the hippocampus commences with SUB (inferior) and CA1 (superior). Progressing posteriorly, the SUB travels medially, the lateral border between it and CA1 being oblique in nature (West and Gundersen, 1990). CA3 and DG appear as segmentation progresses toward the body of the hippocampus, with two portions of DG initially appearing due to the folding of the hippocampal head. In line with previous reports (West and Gundersen, 1990; Amunts et al., 2005), the hippocampal–amygdaloid transition area (HATA) was treated as a separate region and was therefore not included in our analysis.

As described in Duvernoy (2005), when viewed coronally the beginning of the hippocampal tail resembles the body of the hippocampus, and it is only in the middle portions of the tail that it first starts to broaden (posterior, Figure 2) and then narrows to disappear behind the splenium.

**Figure 3:**
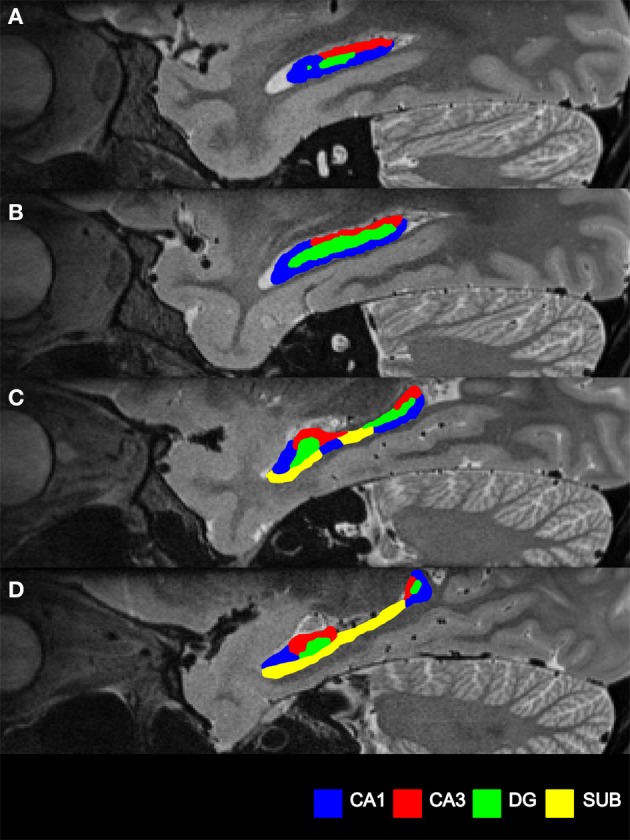
**Subfield segmentation in the sagittal plane. (A)** Sagittal section through an averaged T2-weighted image of the hippocampus of a participant. Blue indicates CA1, red indicates CA3, the DG in green becoming visible. **(B)** Proceeding medially through the hippocampus. **(C)** The body starts to thin and subiculum (yellow) replaces part of CA1. **(D)** Only head and tail remain at the medial end of the hippocampus, with now most of the subiculum in view.

After this phase of segmentation was completed, the view was rotated to the sagittal plane to confirm and refine the segmentation. In the sagittal view, at the lateral-most edge CA1 is observed first. Continuing medially across the hippocampus, CA3 then appears (superiorly) as well as CA1 (inferiorly), gradually revealing the DG sandwiched between CA1 and CA3 (Figures 3A,B). Continuing medially, the SUB finally emerges (Figures 3C,D), replacing some of CA1 in the head and body of the hippocampus.

**Figure 4:**
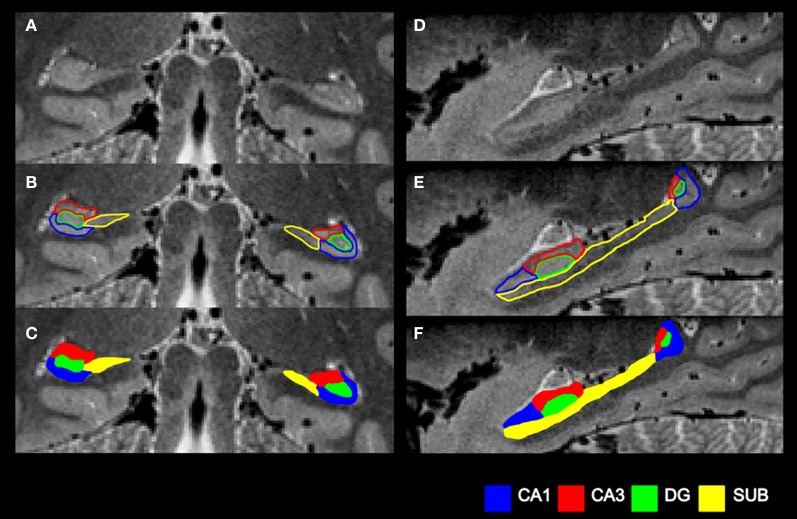
**Summary of the subfield segmentation procedure. (A)** An example coronal slice before segmentation. **(B)** The same coronal slice with the subfield boundaries demarcated. **(C)** The final subfield segmentation of this coronal slice. **(D)** An example sagittal slice before segmentation. **(E)** The same sagittal slice with the subfield boundaries demarcated. **(F)** The final subfield segmentation of this sagittal slice.

**Figure 5:**
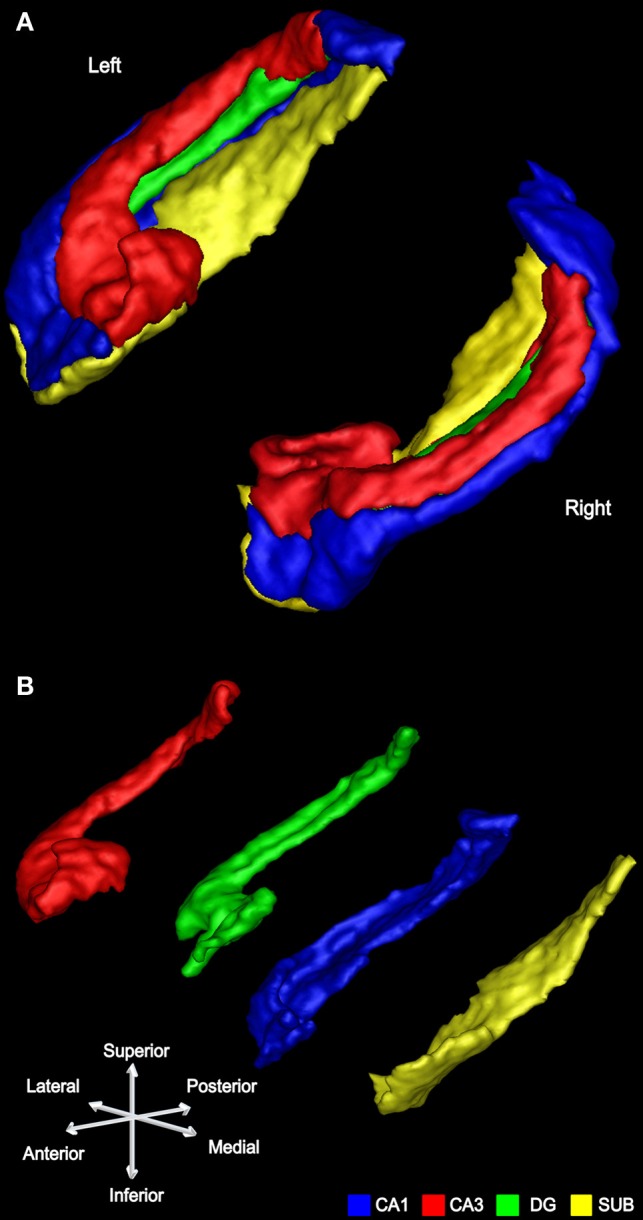
**An example of subfield segmentation in 3D. (A)** The left and right hippocampus of a participant shown in 3D with the subfields conjoined. **(B)** The same left hippocampus is shown with its subfields separated.

These manual segmentations (see also Figure 4) generated a set of regions of interest (ROIs) for each participant in each hemisphere: CA1, CA3, DG, and SUB (Figure 5). The average amount of time taken to segment the subfields of one hippocampus was approximately two days. Intra-rater and inter-rater reliabilities were calculated using the Dice overlap metric (Dice, 1945), defined as the volume of overlap between two ROIs, divided by the mean volume. As in other subfield segmentation studies (Van Leemput et al., 2008; Yushkevich et al., 2009; Malykhin et al., 2010), five consecutive slices located in the body of the hippocampus were chosen. Intra-rater reliability was assessed by comparing two sets of segmentations by HMB with a 6 months interval between segmentations. Inter-rater reliability was assessed by comparing the segmentations of HMB and MJC. All ROIs used in the MVPA analysis were delineated by HMB.

### Image preprocessing

SPM5 was used for image preprocessing. The first six functional volumes were discarded to allow for T1 equilibration (Frackowiak et al., 2004). The remaining functional volumes were spatially realigned to the first image of the series, and distortion corrections were applied based on the field maps using the unwarp routines in SPM (Andersson et al., 2001; Hutton et al., 2002). Each participant's whole brain MT FLASH structural scan was then co-registered to a mean image of their realigned, distortion-corrected functional scans. Following this, the high-resolution T2-weighted averaged structural image was co-registered to the MT FLASH structural scan, bringing all images into alignment (this co-registration was performed prior to the manual segmentation of the subfields). Functional data were minimally smoothed with a 3-mm FWHM Gaussian kernel (as in Bonnici et al., 2012). Each trial was modeled as a separate regressor, where the time of display of each stimulus was modeled as an event and convolved with the canonical hemodynamic response function. Participant-specific movement parameters were included as regressors of no interest. Participant-specific parameter estimates pertaining to each regressor (betas) were calculated for each voxel. The voxel size used by the classifier was that of the fMRI scans, namely 1.5 × 1.5 × 1.5 mm^3^; the mean number of voxels in each subregion was: CA1: 267.06 (SE 66.77), CA3: 248.47 (SE 62.12), DG: 183.16 (SE 45.79), and SUB: 111.13 (SE 27.78).

All data were analyzed in the native space of each participant, using the participant-specific ROIs. Normalization was not required as we did not need to align the subfields across participants in this MVPA context.

### MVPA

There are a number of different methods available for MVPA (reviewed in Chadwick et al., 2012). Here we used a two-step procedure incorporating first feature selection and then final multi-voxel pattern classification (Guyon and Elisseeff, 2003). The classification procedure involved splitting the fMRI data into two parts: a “training” set used to train a linear support vector machine (SVM; Duda et al., 2001) with fixed regularization hyperparameter (*C* = 1) in order to identify response patterns related to the stimuli being discriminated, and a “test” set used to independently test the classification performance. Trials on which participants rated their decisions as “fairly sure” and “very sure” were used for all classifications. Prior to classification, feature selection was carried out on the data from *the training set only* (guaranteeing that the final classification process would be independent from the feature selection, thus avoiding “double dipping,” Kriegeskorte et al., 2009).

The purpose of feature selection is to reduce the set of features (in this case, voxels) in a dataset to those most likely to carry relevant information. This is effectively the same as removing voxels most likely to carry noise, and is a way of increasing the SNR. Feature selection was implemented using a multivariate searchlight strategy (Kriegeskorte et al., 2006), which examines the information in the local spatial patterns surrounding each voxel within the search space. Thus, for each voxel within an ROI, we investigated whether its local environment contained information that would allow accurate decoding, for example, of the two scenes. For a given voxel, we first defined a small sphere with a radius of three voxels centred on the given voxel. This radius was chosen because previous demonstrations of hippocampal decoding using the searchlight method used radius three (Hassabis et al., 2009; Chadwick et al., 2010; Bonnici et al., 2012). Note that the spheres were restricted so that only voxels falling within the given region of interest were included. Therefore the actual shape of the sphere, and the number of voxels within it, varied depending on the proximity to the region of interest's borders. This procedure then allowed the selection of the searchlight voxel sets that contained the greatest degree of decoding information within the training dataset, using a k-fold cross-validation procedure, where k equaled the number of experimental trials minus the trial left out for the final classification. The average number of voxels selected by the searchlight and used in the SVM for each subregion was: CA1: 164.15 (SE 12.63), CA3: 156.58 (SE 12.10), DG: 132.54 (SE 5.54), and SUB: 81.58 (SE 7.39).

Using the voxel subset obtained from the feature selection procedure, the SVM classifier was then trained to discriminate between the two scenes using the training image dataset, and tested on the independent test dataset. The classification was performed using the LIBSVM software (Chang and Lin, 2011).

### Data analysis

The classifier accuracy values for each brain region were compared to chance (50%) using *t*-tests. Comparisons of classifier accuracy values between regions were conducted using repeated measures ANOVAs and any significant effects were further interrogated using paired *t*-tests. A threshold of *p* < 0.05 was employed throughout.

## Results

### Behavioural data

**Figure 6:**
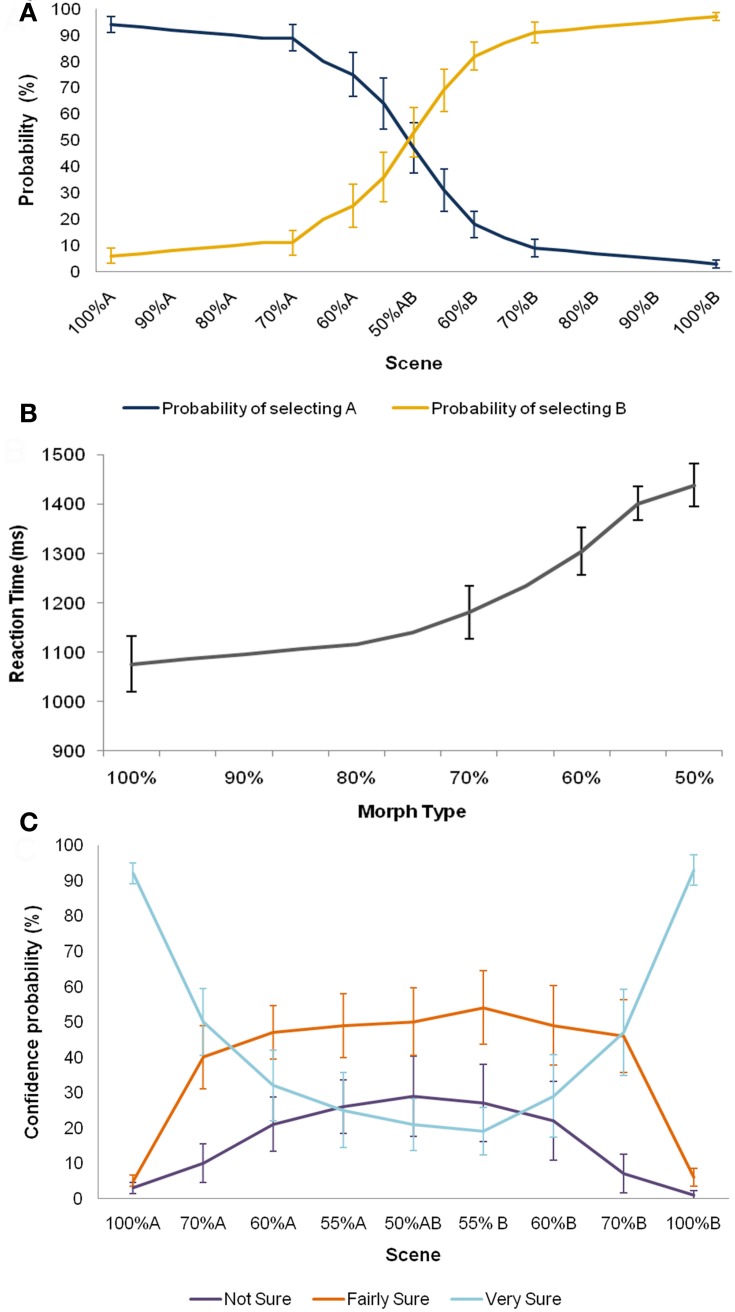
**Behavioural data.** Means ± 1 SE are shown. **(A)** The psychometric function for accuracy for the 16 participants showed a sigmoid profile. **(B)** Participants were less accurate and slower with increasing noise in the sensory input. **(C)** Participants' pattern of confidence ratings also followed the expected distribution. Morphs approaching the two original scenes were afforded higher confidence ratings, and more ambiguous morphs lower ratings.

These data are reported in Bonnici et al. (2012) but are summarised here and in Figure 6 for convenience. Prior to scanning, participants learnt to select the appropriate action for each scene (A, B), taking an average of 5.5 trials (SD 5.97) to reach criterion (10 correct responses in a row). To ensure that choice performance had stabilized before scanning, each participant performed at least 20 trials during this phase. Participants then received practice on the scene morphs task, in order to familiarize them with each of the seven morph scenes, and to ensure that behavioral performance had stabilized before scanning (see “Materials and Methods”).

During scanning, participants viewed both original scenes (100% A, B), as well as the seven morph scenes a total of 40 times each, randomly intermixed. Whilst participants were not provided with feedback during scanning, they were instructed to choose the action most likely to yield reward given the composition of the scene being viewed, and rate their level of confidence in their choice. The psychometric function for accuracy for the 16 participants showed a sigmoid profile (Figure 6A). Further, participants were slower and less accurate with increasing noise in the sensory input (Figure 6B), consistent with previous suggestions that decisions under perceptual uncertainty reflect the accumulation of evidence toward a threshold (Gold and Shadlen, 2007). Participants' pattern of confidence ratings also followed the expected distribution. Morphs approaching the two original scenes were afforded higher confidence ratings, and more ambiguous morphs lower ratings (Figure 6C). Of note, even when the perceptual input was entirely ambiguous (i.e., 50% morphs), participants tended to rate their decisions with a moderate degree of confidence (i.e., “fairly sure” or “very sure”), on average, rather than a subjective sense of guessing. Behavioural accuracy (*p* = 0.40), reaction times (*p* = 0.19), and confidence ratings (*p* = 0.35) did not change significantly over the course of scanning.

Following the scanning session, participants took part in a post-experimental testing session which provided ancillary information concerning the nature of the strategies used during the discrimination task (see “Materials and Methods”). This revealed that in general participants were able to select the correct action in response to rotated versions of the original scenes suggesting that behavioral performance was based on view-independent scene representations, rather than the sampling of individual features (correct scene selection mean: 33/40; SD 5.03). All but two participants performed significantly above chance on this task. When these two participants were removed from the analyses described below, there was no change to any of the findings. In addition, all participants were able to draw the main features of scenes A and B, and could indicate the differences between the two.

### Hippocampal subfield segmentation

The Dice metric results were generally high, indicating that the scanning protocol was suited to reliable delineation of the hippocampal subfields. Intra-rater reliability was: 0.86 for CA1, 0.72 for CA3, 0.79 for DG, and 0.7 for subiculum. Inter-rater reliability was: 0.80 for CA1, 0.74 for CA3, 0.80 for DG and 0.57 for subiculum.

### MVPA

Each classifier produced an accuracy value for each region of interest in each hemisphere in every participant. For every analysis and region, a comparison between the accuracy values in the left and right hemisphere was conducted using a one-way repeated-measures ANOVA. None of these tests demonstrated any significant hemispheric differences, and therefore all results reported are collapsed across hemispheres.

**Figure 7:**
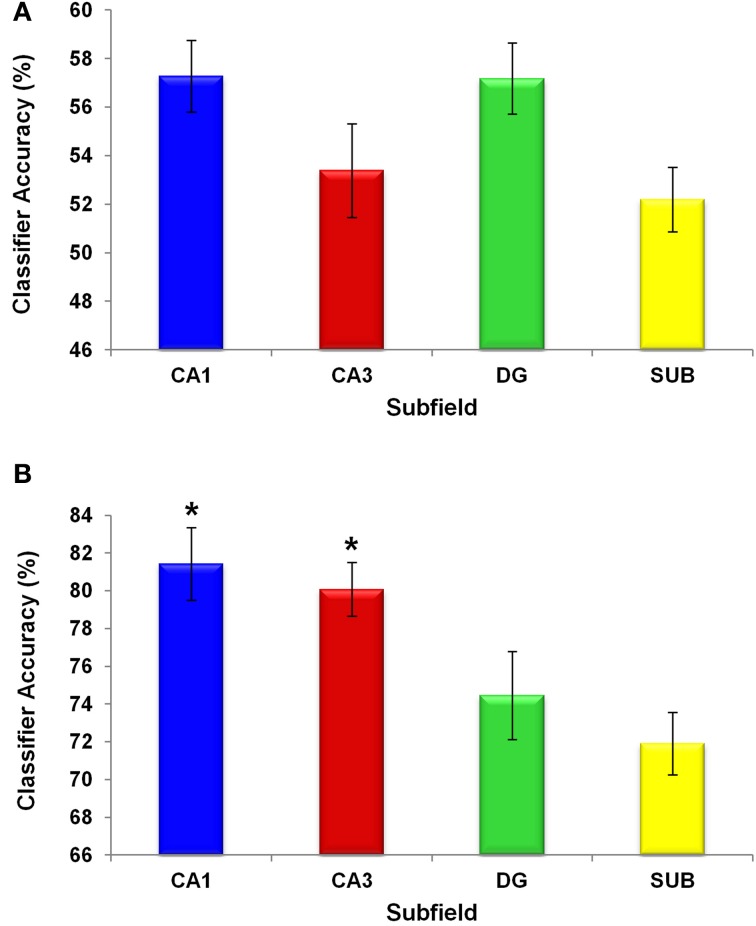
**Classifier performance in hippocampal subfields.** Chance was 50%. Means ± 1 SE are shown. All subfield classifier performances were significantly above chance. The y axis shows the percentage accuracy of the classifiers **(A)** under perceptual certainty, when discriminating between the 100% A and 100% B scenes, and **(B)** under perceptual ambiguity, when making decisions about the 50% morphed scenes. Under perceptual ambiguity classifiers operating on voxels in both CA1 and CA3 showed significantly better performance (^*^p < 0.05) than those operating on voxels in DG and subiculum.

We first asked whether patterns of activity in the hippocampal subfields distinguished between the two original scenes, providing evidence for the coding of scene-specific information in these regions. We carried out an MVPA analysis in which a classifier for each ROI was trained on part of the 100% A and 100% B scene trials, labeled according to participants' choices. The classifiers' performance was then tested on an unseen portion of trials (see “Materials and Methods”). Each subfield classifier was able to distinguish between the two scenes significantly above chance [CA1: *t*_(15)_ = 5.22, *p* = 0.0001; CA3: *t*_(15)_ = 2.63, *p* = 0.019; DG: *t*_(15)_ = 5.67, *p* = 0.0001; SUB: *t*_(15)_ = 2.17, *p* = 0.046; Figure 7A]. No significant differences between the subfields were observed [*F*_(3, 36)_ = 2.638, *p* = 0.064]. This shows that information about the scene currently being experienced is present in all subfields, under conditions of perceptual certainty, and the above-chance decoding suggests that it is possible to deploy MVPA in the subfields of the hippocampus.

Given that participants were performing a decision task, a key question, however, is whether these patterns of activity comprise neural representations of the currently viewed scene (i.e., scene A), or instead retrieved motor actions (e.g., right button press). To address this issue, as in Bonnici et al. (2012) we again trained a classifier in each ROI to distinguish between the 100% A and 100% B trials, labeled according to participants' decisions. Once training was complete, the classifier was then tested on the 50% morph trials, also labeled according to participants' choices. Thus, the classifier was tested to see if it could distinguish between the participants' decisions on whether the 50% morph trials were more similar to the 100% A scene or the 100% B scene. If retrieved motor actions drive the ability of the classifier to decode participants' decisions in relation to 100% scenes, one would predict that a classifier trained on 100% scenes would perform similarly when tested on 50% morph trials. In fact, classifier accuracies in the subfields were not significantly different from chance [CA1: *t*_(15)_ = 1.35, *p* = 0.202; CA3: *t*_(15)_ = 0.567, *p* = 0.581; DG: *t*_(15)_ = −1.83, *p* = 0.091; SUB: *t*_(15)_ = −0.008, *p* = 0.994], making it unlikely that motor variables contributed significantly to decoding of the currently viewed scene.

Having obtained evidence that the hippocampal subfields support distinct scene representations where perceptual input is complete, we next turned our attention to the data during trials where morph scenes were viewed. We focussed on the 50% morph trials, where the perceptual properties of the stimulus were equidistant from both of the original scenes. Behaviourally, the participants tended to categorise these morphs as scenes A and B equally often (Figure 6) but interestingly, as noted above, these choices were accompanied by a relatively high level of confidence in the decisions (i.e., “fairly sure” or “very sure”), suggesting that they were not merely guesses. This is important, because it permitted us to investigate whether there was any information in the hippocampal subfields that allowed us to differentiate the decision states A and B when the visual properties of the stimulus were exactly matched (i.e., it was always the same 50% morph stimulus). If there were distinct patterns of activity for these decision states, then this would provide evidence for a pattern completion process, whereby a perceptually ambiguous stimulus was “pattern completed” into one of two decision categories, leading to a participant confidently asserting that the stimulus belongs to one category over the other. A classifier for each ROI was trained on part of the 50% morph scene trials, which were labeled according to participants' choices. The classifier's performance was then tested on an unseen portion of 50% morph trials. Each subfield classifier was able to classify these trials significantly above chance [CA1: *t*_(15)_ = 16.352, *p* = 0.0001; CA3: *t*_(15)_ = 21.257, *p* = 0.0001; DG: *t*_(15)_ = 10.472, *p* = 0.0001; SUB: *t*_(15)_ = 13.246, *p* = 0.0001; Figure 7B].

We also looked for differences between the subfields using a one-way repeated measures ANOVA and found a significant regional difference [*F*_(3, 36)_ = 6.842, *p* = 0.001], where classification was significantly more accurate in CA1 and CA3 compared with DG and SUB [Figure 7B; CA1>DG: *t*_(15)_ = 2.385, *p* = 0.034; CA1>SUB: *t*_(15)_ = 3.441, *p* = 0.005; CA3>DG: *t*_(15)_ = 3.178, *p* = 0.008; CA3>SUB: *t*_(15)_ = 4.291, *p* = 0.001; CA1>CA3: *t*_(15)_ = 0.609, *p* = 0.554; DG>SUB: *t*_(15)_ = 0.899, *p* = 0.386].

## Discussion

The aim of this study was to perform MVPA in the hippocampal subfields. In order to do this, we first had to devise a means of segmenting the subfields, with the provisos that we wanted to include subfields throughout the whole hippocampus, and to differentiate CA3 and DG. Examining fMRI data from an established paradigm (Bonnici et al., 2012) that had relevance for computations purported to occur in hippocampal subfields, we found that it was possible to decode patterns of fMRI activity across voxels significantly above chance in all subfields (CA1, CA3, DG, subiculum) and predict which scene stimuli were being perceived, under conditions of both perceptual certainty and ambiguity. In the latter case, where pattern completion was dominant, classifiers operating on patterns of voxels across both CA1 and CA3 achieved particularly high accuracy. The good intra- and inter-rater reliability scores for the segmentation of the subfields from the high-resolution structural MRI scans, the above-chance fMRI decoding across subfields, particularly those revealing differential effects consistent with the mechanisms proposed to be at work there, suggest MVPA in human hippocampal subfields is possible and informative.

In order to truly elucidate the role of the hippocampus, an understanding of the functions of its subfields is required. Currently, identifying human hippocampal subfields *in vivo* from structural MRI scans is a significant challenge, such that there is no widely agreed method for their segmentation. In the main, current protocols are limited in the extent of hippocampal tissue they consider (often ignoring subfield distinctions in the head and tail, and being unable to differentiate between CA3 and DG). Thus, important functional distinctions within the hippocampus could be missed. To ameliorate these problems, for each participant we acquired four T2-weighted structural MRI scans with 0.5 mm isotropic resolution. The average of these images, with improved SNR, provided enhanced subfield contrast throughout the whole structure, and in particular permitted identification of the elusive boundary between CA3 and DG. Subfield delineation was reliable both within and between the experimenters who performed the segmentations.

We therefore believe that our scan sequence and resultant subfield segmentation protocol represents an improvement on extant procedures offering, with a standard 3T clinical whole-body MRI scanner, the opportunity for a more complete investigation of each of the major subfields separately throughout the whole hippocampus. However, there is also a disadvantage to our approach. In the first instance, the time taken to acquire the four T2-weighted structural scans is 48 min per participant. In our experience, averaging across fewer than four scans adversely affects SNR and the ability to discern critical subfield boundaries. On top of this, the manual segmentation of the subfields by an experienced experimenter takes up to two days per hippocampus. Thus, for the current study with 16 participants, subfield delineation took over 2 months for one experimenter, with additional time for the repeat measurements by this person, and then a further period of segmentation by the second rater. While this resulted in complete and accurate subfield identification, clearly in studies with large numbers of participants, this would not be practical. It is therefore essential that the development of automated subfield segmentation continue to be pursued, but this must include the whole hippocampus (not just the body), all major subfields being delineated separately, and basing the segmentations on well-established and agreed anatomy of the hippocampal subfields (e.g., West and Gundersen, 1990; Duvernoy, 2005; Yushkevich et al., 2009).

Having delineated the hippocampal subfields, we were then able to deploy MVPA in this context. We used an existing task that likely had relevance for the computations that operate in the hippocampal subfields (Bonnici et al., 2012). We found that information about two highly similar scenes was present in all of the subfields and this permitted above-chance prediction by the classifiers of which scene was being perceived. That there was no significant difference between subfield classifier performances may initially appear surprising given that the role of the DG and CA3 in pattern separation is often emphasized (Leutgeb et al., 2007; Leutgeb and Leutgeb, 2007). However, in our experiment the participants were familiarized with the scene and morph stimuli prior to being scanned (as the MVPA approach we employed depends on stable representations on a trial-by-trial basis). As such, while sparse coding in the DG may be an initial step in the generation of orthogonalised codes, this part of the process was not scanned here. Instead we observed the subsequent expression of these pattern-separated representations in successive stages of hippocampal processing (i.e., in CA3 and then CA1; McClelland et al., 1995).

In a second analysis we focussed on trials where the perceptual input was equidistance from the two original scenes (50% morphs). Interestingly, despite the stimuli being identical on these trials when they occurred throughout the experiment, behaviourally the participants tended to categorise these morphs as scenes A and B equally often, and these choices were accompanied by a relatively high level of confidence in the decisions, suggesting that they were not merely guesses. Using the participants' decisions to label the trials as either scene A or scene B, we again observed all subfields performing significantly above chance. On this occasion, a significant difference in classifier performance emerged between the subfields, where CA1 and CA3 classifiers were significantly more accurate than the DG and SUB classifiers. Because of the perceptual uncertainty on these 50% morph trials, the participants were required to rely on internal representations of the original scenes in order to make their decision. This may require initial retrieval of similar patterns by pattern completion, which is thought to occur in CA3 (Nakazawa et al., 2002; Leutgeb et al., 2004; Leutgeb and Leutgeb, 2007; Gilbert and Brushfield, 2009) and then comparing these internal representations in order to reach a decision. It has been suggested that this comparison process may occur in CA1 (Kumaran and Maguire, 2007; Chen et al., 2011). Of particular note here is the classifier performance difference between the CA3 and DG subfields. Although both classifiers produced accuracy results significantly above chance, the result from the CA3 classifier was significantly more accurate than that obtained from the DG classifier. To the best of our knowledge, results showing functional differentiation between CA3 and DG using fMRI have not been reported, and our finding underscores the need for future studies to segment and study these subfields separately.

In conclusion, by using a high-resolution T2-weighted structural MRI scanning protocol we were able to improve on extant hippocampal subfield segmentation approaches by delineating subfields in the whole hippocampus, and separating CA3 from DG. We hope that this will be useful for those interested in studying the subfields in a range of contexts, in relatively small groups of participants. We have also shown that MVPA in the subfields is possible and informative, opening up new opportunities to examine how different types of information (e.g., spatial, autobiographical) are represented and processed at this fundamental level.

### Conflict of interest statement

The authors declare that the research was conducted in the absence of any commercial or financial relationships that could be construed as a potential conflict of interest.
